# Effect of Calcium Channel Blockers versus Diuretics for Hypertension

**DOI:** 10.1155/2022/3739463

**Published:** 2022-08-11

**Authors:** Yi Chunxue, Zhang Jianming, Wang Jianbing

**Affiliations:** Chongqing University, Three Gorges Hospital, Chongqing, China

## Abstract

**Background:**

Although calcium channel blockers (CCBs) are recognized as clinical first-line agents for the treatment of hypertension, their use in combination with diuretics in cardiovascular disease caused by hypertension remains controversial.

**Methods:**

We searched the three major databases of the Cochrane Library, EMBASE, and PubMed for the terms “calcium channel blockers,” “thiazide diuretics,” “loop diuretics,” and “hypertension,” “randomized controlled trials” and “meta-analysis trials.” These terms were searched from January 1991 to October 2021.

**Results:**

For the primary outcome, in 5 studies including 35,057 patients, there was no statistically significant difference in all-cause mortality with calcium channel blockers compared with diuretics (*RR* = 0.98, 95% CI 0.92–1.04, *I*^2^ = 0). In four studies including 33,643 patients with major cardiovascular events, there was no statistically significant difference in major cardiovascular events with calcium channel blockers compared with diuretics (*RR* = 1.00, 95% CI 1.04–1.09, *I*^2^ = 0).

**Conclusion:**

There is no statistically significant difference between calcium channel blockers and diuretics in terms of cardiovascular clinical prognosis in hypertensive patients, but there are positive implications for clinical guidance, which need to be fully validated in new large randomized controlled trials.

## 1. Introduction

Hypertension is a common disease in middle-aged and elderly people, not only as a cardiovascular disease in itself but also as a potential risk for other geriatric diseases in middle-aged and elderly patients, especially coronary heart disease, diabetes mellitus, and stroke [1–3]. Hypertension is a clinical syndrome characterized by increased arterial blood pressure (systolic and/or diastolic) in the body circulation (systolic blood pressure ≥140 mm Hg and diastolic blood pressure ≥90 mm Hg), which may be accompanied by functional or organic damage to the heart, brain, kidneys, and other organs. Hypertension is the most common chronic disease and the most important risk factor for cardiovascular and cerebrovascular diseases. Blood pressure in normal individuals fluctuates within a certain range in response to changes in the internal and external environment. In the overall population, blood pressure levels rise gradually with age, more markedly with systolic blood pressure, but after age 50, diastolic blood pressure shows a decreasing trend and pulse pressure increases. Its incidence and disability have increased dramatically since the 1990s, and its corresponding treatment deserves our utmost attention [4].

In the treatment of hypertension, the principle of “early, timely, and combined use” is well established, and calcium channel blockers are usually used as first-line agents in combination with other antihypertensive drugs, such as angiotensin-converting enzyme (ACE) inhibitors, angiotensin receptor blockers (ARBs), and *β*- and *α*-blockers. Diuretics have been less studied and they are usually used in patients with renal insufficiency [5–7]. This study is considered an important clinical guideline. This study was searched and screened to include a series of high-quality randomized controlled trial studies to objectively assess and analyze the clinical efficacy of calcium channel blockers versus diuretics in hypertension in order to obtain an objective basis for more and better treatment options to achieve better outcomes.

## 2. Methods

### 2.1. Study Registration

We used the methods recommended by the Cochrane Collaboration to conduct the meta-analysis. The methods used to report the study followed the Preferred Reporting Items for Systematic Reviews and Meta-Analyses 2020 (PRISMA) statement and are registered with PROSPERO [8]. Meta-analysis is a statistical method used to compare and synthesize the results of studies addressing the same scientific question; the significance of the conclusions depends on the quality of the included studies and is often used for quantitative merger analysis in systematic reviews. By integrating all relevant studies, the effects of healthcare can be estimated more precisely than those of individual studies, and it facilitates the exploration of the consistency of evidence across studies and the variability between studies. When the results of multiple studies are inconsistent or none of them are statistically significant, meta-analysis can be used to obtain statistical analysis results that are close to the true picture.

### 2.2. Eligibility

We included trials that met each of the following PICOS criteria:

1. Subjects were all patients (≥50 years) with confirmed hypertension (baseline blood pressure (BP) of at least 140 mmHg systolic or 90 mmHg diastolic, measured in a standard way, at least two measurements according to standard methods)
2. Intervention: calcium channel blockers were given to all experimental groups of the study (no restrictions on specific drugs, dosing regimen, mode of administration, or duration of observation)
3. Comparison intervention: diuretics: all included study controls can be either placebo controls or blank controls
4. Outcomes: primary outcomes: all-cause mortality, stroke (nonfatal and fatal stroke), and major cardiovascular events
5. Study design: RCTs

### 2.3. Search Strategy

We set up search strategies based on keywords. These include PubMed, Embase Database, and The Cochrane Library, and the search period was from January 1991 to October 2021. It is a comprehensive collection of clinical randomized controlled trials (RCTs) related to hypertension and diuretic. The search was set up as a subject search, with the following terms: “calcium channel blockers,” “thiazide diuretic,” “loop diuretic,” “hypertension,” “randomized controlled trial,” and “meta-analysis trials;” references to the included literature were also searched and browsed, thus to broaden the search and minimize omissions.

### 2.4. Study Selection

Two reviewers (Chunxue YI and Jianbing Wang) independently reviewed the titles and abstracts of potentially relevant studies as well as the full text; any disagreement between the two reviewers was resolved by discussion and decision by a third reviewer (Jianming Zhang).

### 2.5. Assessment of Risk of Bias

**Figure 1:**
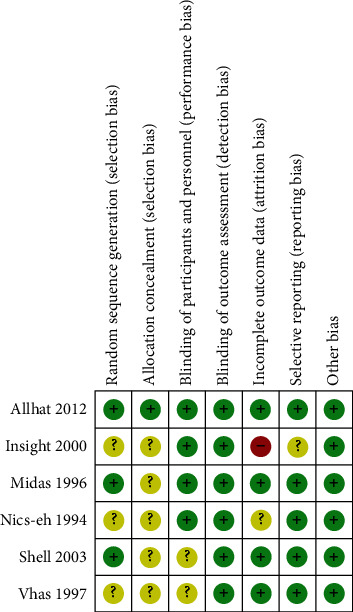
Risk of bias summary.

**Figure 2:**
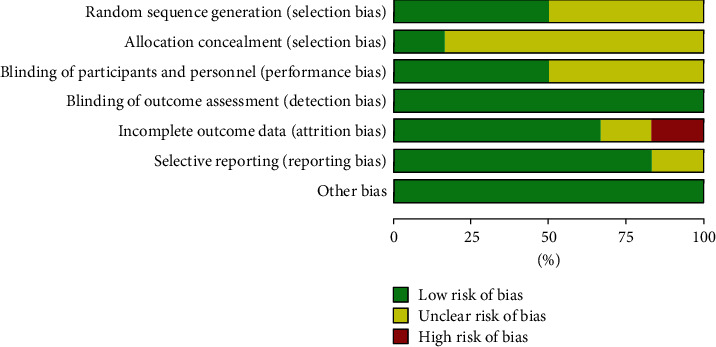
Risk of bias graph.

The Cochrane Handbook for Systematic Reviews of Interventions will be used to assess the quality of reviews. Quality of review: results will be independently cross-checked by two reviewers (Chunxue YI and Jianbing Wang). After internal discussion, any remaining discrepancies are resolved by a third reviewer (Jianming Zhang) [8, 9] (Figures 1 and 2).

### 2.6. Data Synthesis

RevMan version 5.3 (Cochrane Collaboration) will be used for data synthesis. For effect size estimation, RRs and 95% CIs of binarized outcomes will be used. Continuous data will be presented with MD and 95% CI. When different scales of measurement are reported, SMD statistics will be used for the analysis of continuous data. Efforts will be made to identify sources of heterogeneity in each dimension and to provide a narrative and qualitative summary [10–12]. The ensemble composed of thermodynamic systems with the same particle number *n*, temperature *T*, and volume V is called the canonical ensemble. The thermodynamic system of the canonical ensemble must be in a rigid container without any volume change and no material exchange with the environment. However, if the canonical ensemble thermodynamic system has no energy exchange with the outside world, the temperature of the thermodynamic system will convert the kinetic energy and potential energy of its constituent particles to each other and fluctuate. In order to ensure that the temperature of the canonical ensemble thermodynamic system is constant, each scientific system must be in contact with a constant temperature hot bath with a huge heat capacity and a temperature of T. At the same time, in order to ensure that the thermodynamic system and the hot bath are in thermal equilibrium at any time, the heat conduction velocity between them must reach infinity. *M* value is mean, the arithmetic mean. The arithmetic mean, also known as the mean, is the most basic and commonly used kind of average indicator in statistics and is divided into simple arithmetic mean and weighted arithmetic mean. It is mainly applied to numerical data, not applicable to quality data. According to the different forms of expression, the arithmetic mean has different calculation forms and calculation formulas.

## 3. Results

### 3.1. Study Selection

**Figure 3:**
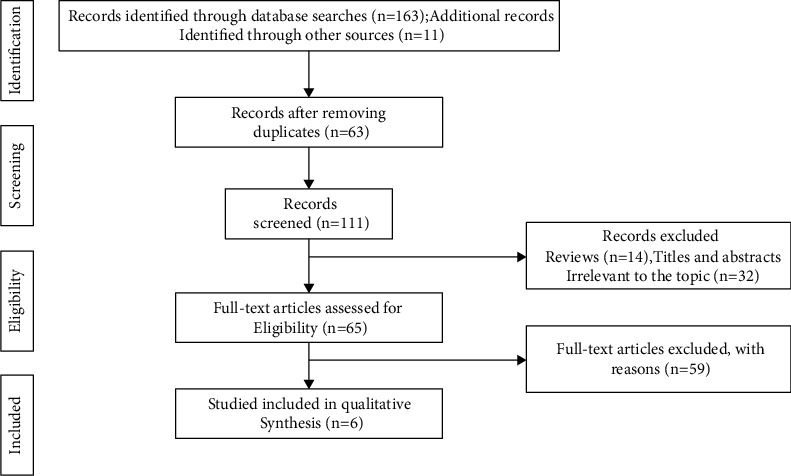
Search strategy and final included and excluded studies.

174 references were searched on the basis of keywords, first excluding 63 duplicate references, then 46 references that did not fit the topic, reading the full text of the literature and excluding 59 references according to the inclusion and exclusion criteria, and finally including 6 references [13]. A flowchart of the literature selection process is shown in Figure 3. The 5 studies are given in Table 1.

### 3.2. Outcomes

**Figure 4:**
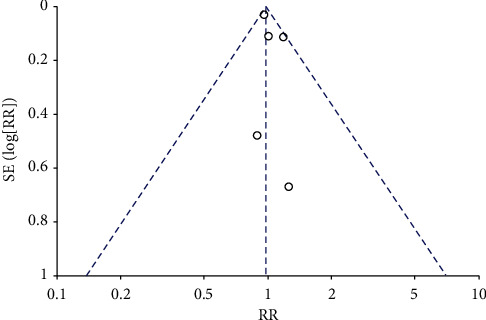
Funnel plot of all-cause mortality.

**Figure 5:**
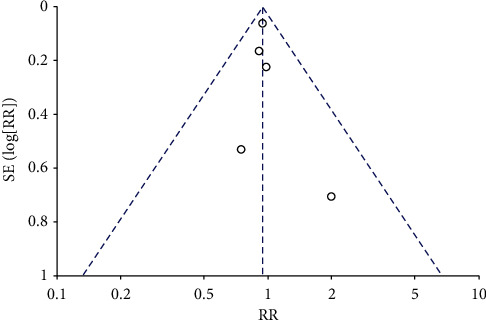
Funnel plot of stroke.

**Figure 6:**
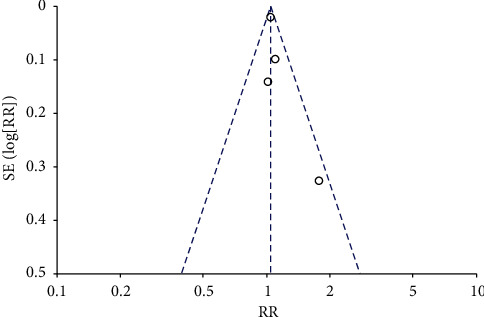
Funnel plot of major cardiovascular events.

For the primary outcome, in 5 studies including 35057 patients, there was not a statistically significant difference in all-cause mortality in calcium channel blockers versus diuretics (*RR* = 0.98, 95% CI 0.92–1.04, *I*^2^ = 0) (Table 2; Figure 4). In terms of secondary outcomes, in 5 studies including 34072 patients in stroke, there was not a statistically significant difference in stroke in calcium channel blockers versus diuretics (*RR* = 0.94, 95% CI 0.84–1.05, *I*^2^ = 0) (Table 3; Figure 5). In 4 studies including 33643 patients in major cardiovascular events, there was not a statistically significant difference in major cardiovascular events in calcium channel blockers versus diuretics (*RR* = 1.00, 95% CI 1.04–1.09, *I*^2^ = 0) (Table 4; Figure 6).

## 4. Discussion

In a systematic meta-analysis of six RCTs in a total of 38,486 patients, we found a better effect of using calcium channel blockers versus diuretics for the treatment of hypertension.

Hypertension is a common cardiovascular disease among middle-aged and elderly patients worldwide, and its progression is closely linked to other geriatric diseases, such as diabetes and stroke, which place a significant financial burden on families [14, 15]. It is therefore particularly important to help prevent or treat this disease. Wright JT's team found that diuretics play a better role in the clinical use of first-line drugs for the treatment of hypertension and can be combined early if the condition requires it. At the same time, however, the use of calcium channel blockers also has a therapeutic effect. In the long term, clinical trials comparing the two will help to understand their clinical efficacy. There were no statistically significant differences in this study between all-cause mortality, stroke, and major cardiovascular events, but there may be some bias in the results as vials are updated in terms of measurement and composition [16, 17].

At present, this review found no statistically significant effect of calcium channel blockers versus diuretics in hypertensive patients, similar to the results of foreign clinical trial studies. This meta-analysis has the following strengths: the meta-analysis focused on a large randomized controlled trial [18–20], which to a certain extent ensures the reliability of the clinical data; and we used the Cochrane Handbook's systematic evaluation method to objectively evaluate the quality of the selected literature and to more comprehensively evaluate the effects of calcium channel blockers and diuretics on hypertensive patients. This systematic evaluation has some limitations as the articles included in this study are dated and further multicentre, multisample clinical trials are needed to validate this study.

## 5. Conclusions

There was no statistically significant difference in cardiovascular clinical prognosis between calcium channel blockers and diuretics in hypertensive patients and no statistically significant difference in major cardiovascular events between calcium channel blockers and diuretics in four studies including 33,643 patients with major cardiovascular events (RR = 1.00, 95% CI 1.04–1.09, *I*^2^ = 0), but with vials in measurement and composition updated in terms of measurement and composition, there may be some bias in the results that need to be validated in a new large randomized controlled trial. This study was searched and screened to include a series of high-quality randomized controlled trial studies to objectively assess and analyze the clinical efficacy of calcium channel blockers versus diuretics in hypertension, so as to obtain an objective basis for more and better treatment options to achieve better outcomes.

## Figures and Tables

**Table 1:** Baseline patient characteristics and treatment parameters by the time of publication in included randomized trials.

Trials	Year	No. of patients	Age (y)	Blood pressure	Dosage (intervention, control)	Outcome
Nics-eh	1994	429	>60	Systolic BP of 160–220 mmHg and diastolic BP < 115 mmHg	CCBs, diuretics	Stroke (nonfatal and fatal stroke)

Midas	1996	883	—	Diastolic BP from 90 to 115 mmHg	CCBs, diuretics	All-cause mortality; stroke (nonfatal and fatal stroke); major cardiovascular events.

Vhas	1997	1414	40–65	Systolic BP ≥ 160 mmHg and diastolic BP ≥ 95 mmHg	CCBs, diuretics	All-cause mortality

Insight	2000	6575	55–80	BP ≥ 150/95 mmHg or systolic BP ≥ 160 mmHg	CCBs, diuretics	All-cause mortality; stroke (nonfatal and fatal stroke); major cardiovascular events.

Shell	2003	1882	—	Systolic BP ≥ 160 mmHg and diastolic BP ≤ 95 mmHg	CCBs, diuretics	All-cause mortality; stroke (nonfatal and fatal stroke); major cardiovascular events.

Allhat	2012	33357	>55	Stage 1 or stage 2 hypertension	CCBs, diuretics	All-cause mortality; stroke (nonfatal and fatal stroke); major cardiovascular events.

**Table 2:** Forest plot of all-cause mortality.

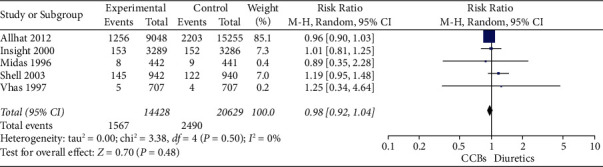

**Table 3:** Forest plot of stroke.

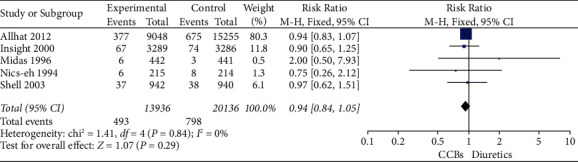

**Table 4:** Forest plot of major cardiovascular events.

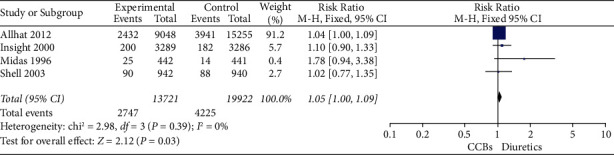

## Data Availability

The data used to support the findings of this study are available from the corresponding author upon request.

## Abbreviations

CI: Confidence interval
RCT: Randomized controlled trial
RR: Risk ratio.
